# Fœtal Monster

**Published:** 1837

**Authors:** 


### ART. VI.—FŒTAL MONSTER.

Our friend and former pupil, Dr. HIRAM PROUT, of *Tuscumbia, Alabama*, has sent to the Museum of the Cincinnati College, a fœtal monster, which, to use the language of his accompanying note, "consists of two human fœtuses, united into one. It has but one head and one body, but the four ears and eight extremities, are distinct and perfect. The faces, breasts and abdomens of the two, are so united as to be invisible, and indeed form one trunk. It presents but two eyes, one on each side, in the line of junction. From its hair and nails, it must have arrived at the 6th month of fœtal existence. Its mother was a negress, and died a few days after her delivery. Its dissection would have been interesting, but I preferred sending it to your museum, as such specimens are rare. I enclose you a drawing of it, made before it had contracted, from the action of the alcohol, in which it is preserved."

We intended to give an engraving from this little drawing, but unfortunately it has been mislaid. We beg Dr. P. to accept our thanks. D.

